# Feasibility of a Serious Illness Communication Program for Pediatric Advance Care Planning

**DOI:** 10.1001/jamanetworkopen.2024.24626

**Published:** 2024-07-26

**Authors:** Danielle D. DeCourcey, Rachelle E. Bernacki, Brett Nava-Coulter, Sithya Lach, Niya Xiong, Joanne Wolfe

**Affiliations:** 1 Department of Pediatrics, Division of Medical Critical Care, Boston Children’s Hospital, Boston, Massachusetts; 2 Division of Psychosocial Oncology and Palliative Care, Dana Farber Cancer Institute, Boston, Massachusetts; 3 Department of Data Science, Dana Farber Cancer Institute, Boston, Massachusetts; 4 Division of Supportive and Palliative Care, Mass General for Children, Boston, Massachusetts

## Abstract

**Importance:**

Pediatric advance care planning (ACP), which aims to ensure care is aligned with family goals and values, is associated with better end-of-life outcomes; however, ACP in pediatrics remains uncommon.

**Objectives:**

To determine the feasibility and acceptability of the Pediatric Serious Illness Communication Program (PediSICP) and explore family-centered outcomes.

**Design, Setting, and Participants:**

This cohort study was a single-group pilot study of the PediSICP in adolescents and young adults (AYAs; age ≥13 y) with serious illness, parents of seriously ill children, and interprofessional clinicians from April 2021 to March 2023 in a quaternary care pediatric hospital. Duration of follow-up was 1 month. Data were analyzed from January 2022 to March 2023.

**Exposure:**

The PediSICP includes clinician training preceding an ACP communication occasion supported by communication guides and a template for electronic medical record documentation.

**Main Outcomes and Measures:**

Outcomes of interest were parent, patient, and clinician experiences with and perceptions of the PediSICP. Feasibility was defined a priori as at least 70% clinician intervention completion rates.

**Results:**

A total of 10 virtual trainings were conducted among 40 clinicians, including 27 physicians, 7 nurse practitioners, 5 nurses, and 1 respiratory therapist, and 30 trained clinicians (75%) conducted and documented 42 ACP conversations with 33 parents (median [IQR] age, 43 [35-51] years; 25 [76%] female) and 5 AYAs (median [IQR] age, 19 [17-19] years; 3 [60%] female) who completed the intervention. The median (IQR) conversation duration was 27 (10-45) minutes. Most clinicians (29 clinicians [97%]) agreed that they felt prepared for the conversation, and all clinicians recommended the PediSICP. Parents reported participation was worthwhile (27 parents [84%]), they felt listened to (31 parents [94%]), and would recommend the PediSICP (28 parents [85%]). Parents endorsed higher therapeutic alliance after the PediSICP intervention compared with before (The Human Connection scale mean [SD] score, 57.6 [6.4] vs 55.3 [7.8]; *P* = .03) and decreased anxiety immediately after the intervention (Generalized Anxiety Disorder–7-item mean [SD] score, 10.1 [7.3] vs 8.4 [6.9]; *P* = .003), which persisted at the 1-month follow-up (mean [SD] score, 7.7 [6.8]; *P* = .03).

**Conclusions and Relevance:**

This pilot cohort study found that the PediSICP was feasible, acceptable, and highly valued by clinicians and parents of children with serious illness. These findings suggest that the PediSICP may empower interprofessional clinicians and improve ACP with families of children and AYAs who are seriously ill.

## Key Points

**Question:**

Is a structured pediatric advance care planning (ACP) intervention feasible and acceptable and is it associated with improved family-centered outcomes?

**Findings:**

In this cohort study including 40 interprofessional clinicians, 36 parents, and 6 adolescents and young adults with serious illness, the structured ACP intervention was both feasible and acceptable. Parents reported higher therapeutic alliance immediately after the intervention and decreased anxiety, which persisted at the 1-month follow up.

**Meaning:**

These findings suggest that this structured advanced care planning intervention was feasible, acceptable, and helpful to clinicians and parents of pediatric patients who are seriously ill.

## Teaser

This cohort study assesses the feasibility and acceptability of a structured pediatric advance care planning program among clinicians, parents of children with serious illness, and adolescents and young adults with serious illness.

## Introduction

Children and adolescents and young adults (AYAs) with serious illness often have a variable clinical course with periods of stability alternating with life-threatening deteriorations; consequently, many children and AYAs experience health crises without opportunities to discuss preferences for medical care. Furthermore, bereaved parents report a lack of preparation to address their child’s medical and emotional needs at end of life (EOL).^1,2^ Advance care planning (ACP) is an iterative process to honor patient and family goals and values involving communication about prognosis and the formulation of care plans addressing symptom management, quality of life, preferences for life-sustaining interventions, and anticipatory guidance about EOL.^3,4,5,6^

Adult studies have found that families who participate in ACP are more satisfied with the care their loved one receives, are more prepared for death, and have improved bereavement outcomes.^7,8,9,10^ Thus far, ACP outcomes research in pediatrics has been primarily focused on adolescents with HIV and cancer, where interventions improve concordance of patient and family preferences with the care they receive and are associated with higher ratings of care quality and decreased decisional regret.^5,6,11,12,13,14,15^ In a 2019 study from our group, ACP was also associated with superior parent-reported EOL care outcomes, including improved preparedness for EOL, the ability to plan their child’s location of death, and superior quality of life for their child.^16^ Additionally, ACP that included specific assessment of family goals was associated with decreases in perceived child suffering at EOL and parental decisional regret.^16^

Despite these benefits and a desire for ACP by both parents and AYAs with serious illness, few generalizable, evidence-based ACP interventions exist, and large scale adoption of pediatric ACP remains unrealized.^5,16,17,18,19,20,21^ Additionally, many pediatric clinicians report both discomfort with and a lack of training in facilitating ACP.^6,17,22,23,24,25^

Multifaceted interventions combining educational and systems-based approaches can increase timely ACP discussions and improve patient and clinician experiences.^10,26,27^ The Serious Illness Communication Guide (SICG) is 1 such tool that has been validated in adults to facilitate effective ACP communication.^26,28,29,30^ However, there is limited research on use of these guides in pediatrics to support clinician-led ACP. To address this gap, the Pediatric Serious Illness Communication Program (PediSICP***)*** was designed using a stakeholder-driven formative research approach.^24^ The PediSICP is a structured ACP intervention consisting of clinician training preceding an ACP communication occasion supported by evidence-based communication guides and a template for electronic medical record (EMR) documentation.^24^ This pilot study aims to evaluate the feasibility and acceptability of the PediSICP with exploration of outcomes associated with program participation, including therapeutic alliance, shared decision-making, communication quality, depression, and anxiety.

## Methods

### Study Design and Population

The protocol for this cohort study was approved by the Boston Children’s Hospital institutional review board. All clinicians and parents provided verbal informed consent to participate, and AYAs provided verbal assent or consent. This study was designed as a mixed-methods, pragmatic, single-group, single-institution feasibility cohort pilot and was conducted at a quaternary care children’s hospital from April 2021 to March 2023. Here, we report on quantitative study results. Reporting followed the Strengthening the Reporting of Observational Studies in Epidemiology (STROBE) reporting guideline for cohort studies. Children and AYAs with serious illness were identified using Feudtner and colleagues definition of complex chronic conditions.^31^ AYAs aged 13 years and older with serious illness and parents of children with serious illness who could read and speak English and had at least 2 readmissions in 1 year or 1 admission lasting more than 2 weeks were eligible to participate. Interprofessional clinicians were eligible if they practiced in an inpatient location or specialty that cares for children and AYAs who are seriously ill.

### Enrollment and Study Procedures

Eligible clinicians were identified through a hospital database, invited once by email to participate, and enrolled from April to December 2021. Parents and AYAs were approached for enrollment between July 2021 and December 2022. A trained research assistant screened for eligible inpatient AYA and parents daily in the intensive care inpatient units and outpatient clinics of enrolled clinicians. For outpatients, participants were also identified through referral from enrolled clinicians. On recruitment, parents and AYAs were provided study information and made aware that an ACP conversation was the primary study intervention. To better understand the unique perceptions of the PediSICP, we did not enroll AYA-parent dyads and enrolled only 1 parent per family; however, both parents could participate in ACP conversations. Trained clinicians were notified when AYAs or parents they were caring for were enrolled. ACP conversations were conducted in-person either during admission with inpatient clinicians at a time convenient for the AYA or parent or at a previously scheduled clinic appointment for outpatient clinicians. Following the conversation, clinicians documented discussion content in the EMR using the PediSICP ACP note template.^24^ All participants received a 1-time retail gift card following intervention completion.

### Clinician Training

In keeping with COVID-19 restrictions, 10 trainings, each with 4 to 8 interprofessional participants, were conducted virtually using Zoom video conferencing software (Zoom Video Communications) from April to December 2021. The 3-hour PediSICP clinician training consisted of a brief didactic session on the value of ACP, demonstration and discussion of the PediSICP, and associated EMR documentation, followed by skills practice using a trained actor along with individual coaching from facilitators with serious illness communication training.^32^

### Data Collection and Survey Instruments

Clinicians completed surveys via electronic data capture before training and after the intervention following their first documented ACP conversation with an enrolled AYA or parent to measure intervention feasibility and acceptability and to explore changes in moral distress using the Moral Distress Scale–Revised (MDS-R).^33^ Preintervention and postintervention AYA and parent surveys were used to measure the intervention’s acceptability using an adaptation of the Lyon Satisfaction Questionnaire, which was developed to assess reactions to an ACP program.^18^ Surveys were also used to evaluate parent exploratory outcomes at 3 time points: before PediSICP intervention (T1), 2 to 7 days after the intervention (T2), and at 1-month follow-up (T3), including parent report of shared decision-making (assessed using CollaboRATE-5,^34^ consisting of 3 items with 5-point Likert responses assessing patients’ perceptions of their involvement in decision-making, with higher scores indicating higher shared decision-making), therapeutic alliance (assessed using The Human Connection [THC] scale,^35^ consisting of 6 items with 4-point Likert responses assessing patient-perceived therapeutic alliance, with scores ranging from 0-64 and higher scores reflecting stronger therapeutic alliance), communication quality (assessed using the Quality of Communication Questionnaire [QOC],^36^ a 17-item survey measuring general and EOL care communication skills on a scale from 0-10 with higher scores indicating higher communication quality), depression (assessed using the Patient Health Questionnaire 9-item scale [PHQ-9],^28^ a 27-point scale where scores of 5, 10, 15, and 20 represent mild, moderate, moderately severe, and severe depression, respectively), and anxiety (measured using the Generalized Anxiety Disorder 7-item scale [GAD-7],^37^ with scores ranging from 0-21 and higher scores indicating higher levels of anxiety, with cut points of 5 considered mild anxiety; 10, moderate anxiety; and 15, severe anxiety).

### Outcome Measures

Our primary aims were to assess feasibility and acceptability. Feasibility was defined a priori as at least 70% of enrolled clinicians completing training and having at least 1 documented ACP conversation with an enrolled AYA or parent using the PediSICP framework. This threshold is consistent with other feasibility studies among parents of children and adults with serious illness.^38,39,40^ We also assessed acceptability of the PediSICP among AYAs, parents, and interprofessional clinicians. Our primary acceptability end point was defined as at least 70% of parent participants would agree or strongly agree that the experience was worthwhile, that they felt listened to, and that they would recommend the intervention to other families, as measured on postintervention surveys. For clinicians, acceptability was defined as at least 70% of postintervention survey respondents would agree or strongly agree that they felt prepared for the ACP conversation, the guide was useful, and they would recommend the PediSICP to colleagues. This pilot study was not designed or powered to test intervention efficacy; however, to collect data for future clinical trials, exploratory outcomes were evaluated at 3 time points, as previously described.

### Statistical Analysis

Self-reported participant characteristics, including race and ethnicity, were collected at enrollment, and reactions to the PediSICP were collected on the post-PediSICP survey and summarized using descriptive statistics. Race was classified as African American or Black, White, or other (including American Indian or Alaska Native, Asian, multiracial, and Native Hawaiian or Other Pacific Islander), and ethnicity was classified as Hispanic or Latino or not Hispanic or Latino. Participants could select multiple races and ethnicities.

To compare participant characteristics by intervention completion status, continuous variables were tested by Wilcoxon rank sum test and categorical variables by Fisher exact test. Wilcoxon signed-rank tests were used to compare differences between instrument scores for exploratory outcomes for parents at 3 time points, as previously described. Given the low number, we did not perform testing to analyze for differences in AYA instrument scores. *P* values were 2-sided and considered statistically significant at *P* < .05. Analyses were conducted using R software version 4.3.1 (R Project for Statistical Computing). Data were analyzed from January 2022 to March 2023.

## Results

### Study Population

**Figure 1.**
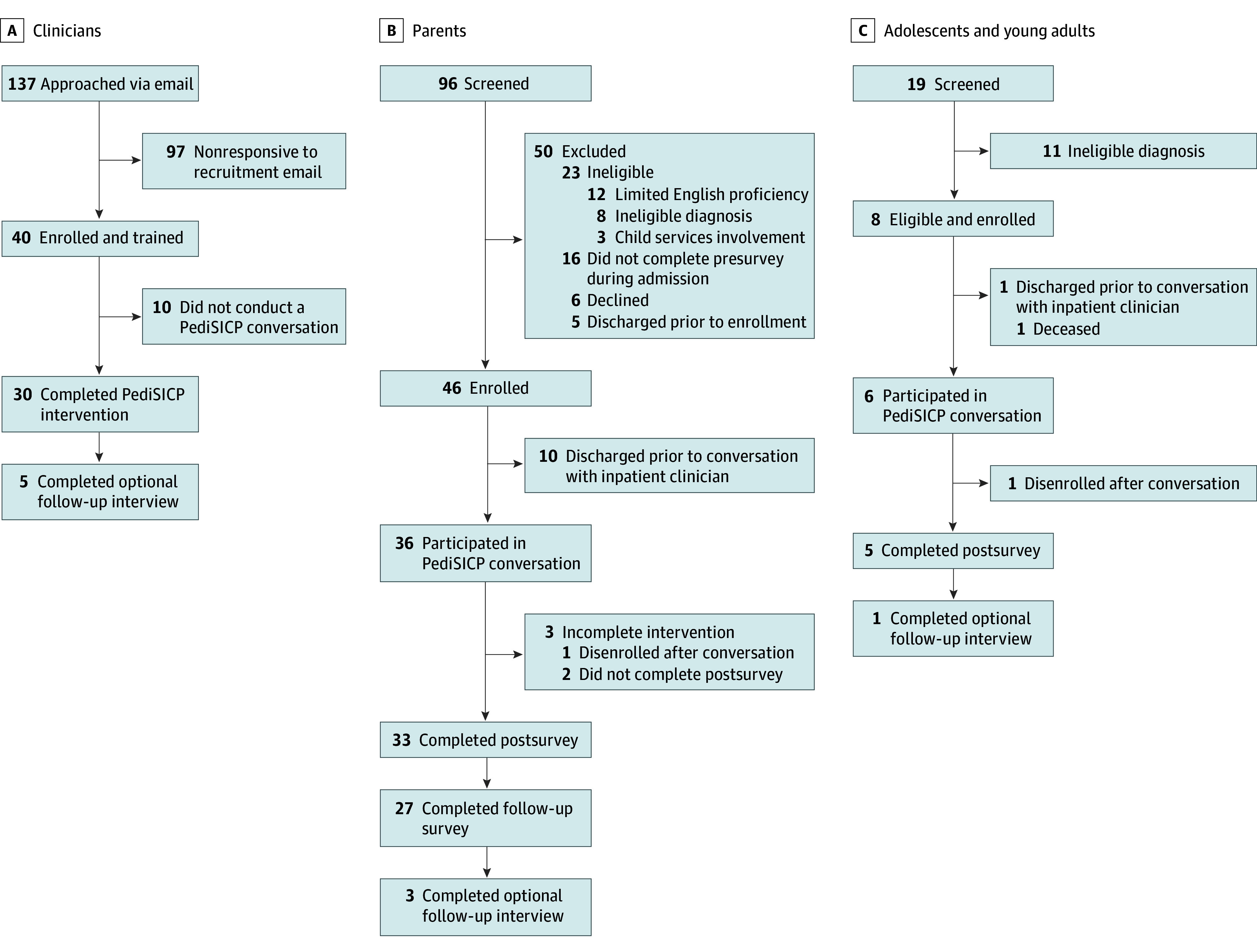
Flowchart of Participant Recruitment to Pediatric Serious Illness Communication Program (PediSICP) Pilot Study

A total of 84 participants provided informed consent and 68 completed the intervention (Figure 1), including 30 clinicians (15 clinicians [50%] with 5-15 years of experience; 24 [80%] female) (Table 1), 33 parents (median [IQR] age, 43 [35-51] years; 25 [76%] female), and 5 AYAs (median [IQR] age, 19 [17-19] years; 3 [60%] female) (Table 2). There was 1 African American or Black clinician (3%) and 29 White clinicians (97%); 1 clinician was Hispanic or Latinx and 29 clinicians (97%) were not Hispanic or Latinx. Most clinicians spoke English as their first language (28 clinicians [93%]) and worked in intensive care (19 clinicians [63%]) (Table 1). Among parents, 2 were African American or Black (6%), 27 were White (82%), and 4 identified as other race (12%); 10 parents (30%) were Hispanic or Latino and 23 parents (70%) were not Hispanic or Latino. Among AYAs, 1 was African American or Black (20%), 4 were White (80%), and none identified as other race; 2 AYAs (40%) were Hispanic or Latino and 3 AYAs (60%) were not Hispanic or Latino (Table 2).

**Table 1.** Characteristics of Interprofessional Clinician Participants Who Completed the Pediatric Serious Illness Communication Program Intervention

Variable	Clinicians, No. (%) (n = 30)
Sex	
Female	24 (80)
Male	6 (20)
Race^a^^,^^b^	
African American or Black	1 (3)
White	29 (97)
Other^c^	0
Ethnicity^a^	
Hispanic or Latino	1 (3)
Not Hispanic or Latino	29 (97)
Degree	
Bachelor’s	3 (10)
≥Master’s	27 (90)
Professional role	
Attending physician	19 (63)
Nurse practitioner	6 (20)
Registered nurse	3 (10)
Resident physician	1 (3)
Respiratory therapist	1 (3)
Provider specialty	
Cardiology	3 (10)
Intermediate and complex care pediatrics	6 (20)
Intensive care	19 (63)
Pulmonary	2 (7)
Experience, y	
<5	10 (33)
5-15	15 (50)
>15	5 (17)
English as first language	28 (93)

^a^ Race and ethnicity are presented as characterized by self-report.

^b^ Multiple selections were allowed.

^c^ Other includes American Indian or Alaska Native, Asian, multiracial, and Native Hawaiian or Other Pacific Islander, based on self-report.

**Table 2.** Demographics of AYA and Parent Participants Who Completed the Pediatric Serious Illness Communication Program Intervention

Characteristics	Participants, No. (%)	
	Parents (n = 33)	AYAs (n = 5)
Sex		
Female	25 (76)	3 (60)
Male	8 (24)	2 (40)
Age, median (IQR), y	43 (35-51)	19 (17-19)
Duration of illness, median (IQR), y	2 (0.9-6.6)	14.6 (13.9-18.9)
Race^a^^,^^b^		
African American or Black	2 (6)	1 (20)
White	27 (82)	4 (80)
Other^c^	4 (12)	0
Ethnicity^a^		
Hispanic or Latino	10 (30)	2 (40)
Not Hispanic or Latino	23 (70)	3 (60)
English as first language	27 (81.8)	5 (100)
Primary diagnosis category		
Congenital or genetic	19 (57.6)	1 (20)
Cardiovascular	6 (18)	0
Metabolic or mitochondrial	2 (6)	0
Neurological	5 (15)	1 (20)
Respiratory	1 (3)	2 (40)
Kidney	0	1 (20)

^a^ Race and ethnicity are presented as characterized by self-report.

^b^ Multiple selections allowed.

^c^ Other includes American Indian or Alaska Native, Asian, multiracial, and Native Hawaiian or Other Pacific Islander, based on self-report.

### Clinician Participation in the PediSICP Training and Feasibility

We contacted 137 clinicians for participation in the PediSICP training; 40 clinicians enrolled in the study by completing the pretraining survey and participating in 1 of 10 virtual trainings (Figure 1). Thirty enrolled clinicians (75%), including 20 physicians, 6 nurse practitioners, 3 registered nurses, 1 respiratory therapist, completed the PediSICP intervention by having and documenting an ACP conversation with an enrolled parent or AYA, thus meeting our feasibility goal. There were no significant differences in clinician characteristics between enrolled clinicians that completed the intervention and those who did not. Reasons for noncompletion were primarily due to the lack of an enrolled AYA or parent during a clinician’s service week or clinic time.

### Parent Participation in PediSICP Intervention

We screened 96 parents, of whom 73 were eligible and 46 enrolled, and 36 enrolled parents participated in an ACP conversation using the PediSICP framework, 33 parents completed the T2 survey, and 27 parents completed the follow-up T3 survey (Figure 1). No significant demographic differences existed between enrolled parents who completed the T2 survey and parents who did not. However, the duration of their child’s illness differed between groups, with a median (IQR) illness duration of 7.3 (0.4-20.2) years for parents completing the intervention vs 2.0 (0.9-6.6) years for those who did not (*P* = .02).

### AYA Participation in the PediSICP Intervention

We screened 19 AYAs, of whom 8 were eligible and enrolled (Figure 1). Six AYAs participated in an ACP conversation using the PediSICP framework, 5 AYAs completed the T2 and T3 surveys. One patient disenrolled after the ACP conversation at their parent’s request.

### Acceptability

**Figure 2.**
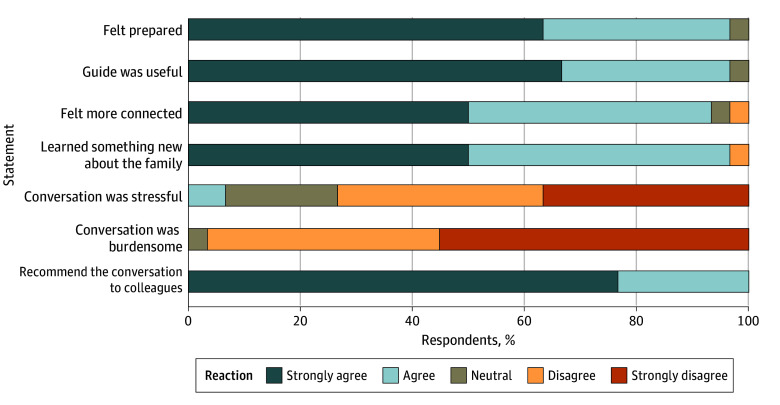
Clinician Reactions to Having an Advance Care Planning Conversation Using the Pediatric Serious Illness Communication Program Framework

Figure 2 details clinician reactions to the ACP conversation using the PediSICP framework. Most clinicians (29 clinicians [97%]) agreed or strongly agreed that they felt prepared for the ACP conversation and the guide was useful; all clinicians reported that they would recommend the framework to colleagues, thus meeting our clinician acceptability targets. Most clinicians (29 clinicians [97%]) agreed or strongly agreed that they learned something new about the family and 28 clinicians (93%) felt more connected to the family after the conversation. Most clinicians (29 clinicians [97%]) disagreed or strongly disagreed that the conversation was burdensome, and 22 clinicians (73%) disagreed or strongly disagreed that the conversation was stressful. The median (IQR) time spent having an ACP conversation was 27 (10-45) minutes.

**Figure 3.**
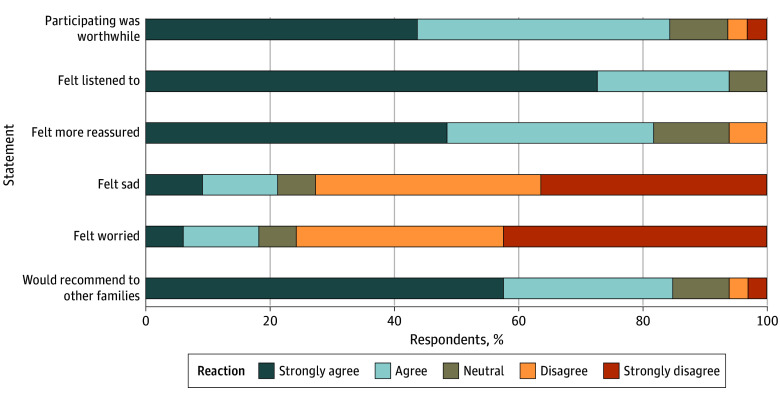
Parent Reactions to Having an Advance Care Planning Conversation Using the Pediatric Serious Illness Communication Program Framework

Of 32 enrolled parents, 27 (84%) agreed or strongly agreed that participation in the PediSICP was worthwhile, 31 (94%) reported that they felt listened to during the ACP conversation, and 28 (85%) would recommend participation to other families (Figure 3), thus meeting our primary acceptability targets. Few parents (7 parents [21%]) reported feeling sad, and 6 parents (18%) reported feeling worried as a result of the ACP conversation; 27 parents (81%) reported feeling more reassured after the conversation.

Most enrolled AYAs also reported that the PediSICP intervention was worthwhile (3 AYAs [60%]), that they felt listened to during the conversation (4 AYAs [80%]), and that they would recommend participation to others (3 AYAs [60%]) while the remaining 40% were unsure.

### Parent and Clinician Exploratory Outcomes

Parents reported significantly higher therapeutic alliance at T2 compared with T1 (HCS mean [SD] score, 57.6 [6.4] vs 55.3 [7.8]; *P* = .03), although this did not persist at the T3 survey (HCS mean [SD] score, 54.8 [8.7]; *P* = .97). There were no significant differences in parent report of shared decision-making at T1 vs T2 (CollaboRATE-5 mean [SD] score, 4.2 [0.6] vs 4.3 [0.6]; *P* = .73) or vs T3 (CollaboRATE-5 mean [SD] score, 4.2 [0.7]; *P* = .36]. Similarly, no significant variation existed at T1 vs T2 in quality of communication assessed with mean QOC scores (mean [SD] score, 8.1 [1.6] vs 8.5 [1.6]; *P* = .25) or vs T3 (mean [SD] score, 8.1 [1.7]; *P* = .99). Parents endorsed moderate anxiety at T1 (mean [SD] GAD-7 score, 10.1 [7.3), which was significantly reduced to mild anxiety at T2 (mean [SD] GAD-7 score, 8.4 [6.9]; *P* = .003) and remained low at T3 (mean [SD] GAD-7 score, 7.7 [6.8]; *P* = .03). Most parents endorsed mild depression at T1 (PHQ-9 mean [SD] score, 7.0 [6.6]), and there were no significant differences at T2 (PHQ-9 mean [SD] score, 7.4 [6.5]; *P* = .76) or T3 (PHQ-9 mean [SD] score, 7.9 [6.7]; *P* = .62) (eTable in Supplement 1). Participating clinicians reported no differences in moral distress before vs after the intervention (MDS-R mean [SD] score, 37.1 [15.8] vs 36.5 [16.9]; *P* = .99).

## Discussion

The results of this single-group pilot cohort study indicate that the PediSICP was both feasible and acceptable to a variety of stakeholders, including interprofessional clinicians and parents of seriously ill children and provide valuable insights into the benefits of utilizing a structured approach to ACP. The PediSICP was developed to provide a structured framework for pediatric clinicians without subspecialty palliative care training to engage in effective and efficient ACP.

In the adult population, the efficacy of ACP and its evidence base have been called into question,^41,42^ and disagreement remains about the value of ACPs in clinical practice and research.^43,44^ While the value of ACP is not as controversial in pediatrics, given evidence of its positive impact,^12,13,14,16,45,46,47^ ACP remains an underused and complex endeavor, as implementation of ACP programs has historically encountered multiple barriers, including clinician discomfort and lack of formal communication training or an evidence-based framework^6,24,25^

A noteworthy finding of this study is the successful implementation of pediatric clinician training using a communication guide as a foundational element, which has been shown to be effective in preparing clinicians for ACP in other subspecialties.^48,49,50,51^ Furthermore, this study targeted interprofessional clinicians, which is both novel and aligned with expert recommendations that stress the need to leverage nonphysicians to guide ACP.^25,43,45^ Another key strength of the PediSICP is its focus on enhancing communication, with most clinicians reporting that they learned something new about and felt more connected to the families they had ACP conversations with, suggesting the program may enhance clinician understanding of family priorities and build trust, which are essential for providing goal-concordant recommendations.

The study also assessed the experiences of parents who engaged in ACP using the PediSICP framework and highlights the potential to enhance patient and family engagement in ACP. Most participating parents reported the intervention was worthwhile and that they would recommend the program to other families, even when these conversations caused sadness or worry. Notably, most parents and AYAs also reported that they felt listened to, which has been shown to build trust and lessen the likelihood of difficult family-clinician relationships^52^ and is essential to ensure value-concordant decision-making, enhance dignity, and relieve suffering.^53,54,55^ Furthermore, our findings are consistent with the broader literature on pediatric ACP, which emphasizes the importance of considering parents’ unique perspectives and preferences in decision-making for their child.^13,16,17,20,21,24,56,57,58^

The PediSICP’s preliminary findings regarding therapeutic alliance and anxiety among parents are particularly encouraging, as these results highlight that although ACP discussions can be stressful for clinicians, they do not cause harm to parents and patients, and in fact, can reduce emotional distress.^19,59,60,61,62^ In inquiring about goals and what is most important, clinicians can strengthen alignment and ensure that patients and families retain some control despite clinical uncertainty.^63,64^ The decrease in anxiety levels observed immediately after the intervention and at the 1-month follow-up, from moderate to mild anxiety, aligns with the adult and pediatric advanced cancer literature, highlighting the potential psychological benefits of ACP for families facing serious illness.^26,28,29,61,62,65^ For therapeutic alliance, to our knowledge, there is no literature in pediatrics describing the minimally important clinical differences in THC scale scores; however, in pediatric advanced cancer populations, similar small statistically significant differences in therapeutic alliance have been reported with advance care planning.^35^ Future research in the form of an adequately powered randomized clinical trial will attempt to improve on the magnitude of this effect and its duration.

### Limitations

This study has several important limitations. The requirement for participants to be English-speaking does not allow for a readily generalizable intervention for diverse populations. Additionally, 97% of the clinicians were White. More research is warranted to understand cultural barriers to ACP, such as race and language, that might impact the program’s feasibility. AYAs were underrepresented, as recruitment was limited owing to high medical burden and a large proportion of AYAs with severe neurologic impairment in our sample. Incorporating the voice of children and AYAs is an important future direction for this work. Because participation was voluntary and successful enrollment of clinicians was based on both self-selection and the ability to attend the training, participants may have been predisposed to feel favorably about the intervention. Additional selection bias may have been introduced by having outpatient clinicians serve as referral gatekeepers for some patients. Given the nature of this pilot study, we did not standardize the time between clinician training and participation in ACP conversations; thus, we were unable to determine whether booster training may be necessary. Furthermore, while we did not detect improvement among some outcome measures, such as quality of communication, as a pilot study, it was intentionally underpowered, and we may have missed identifying improvements in some outcomes. Alternatively, because the QOC questionnaire measures both general communication and communication about EOL care, it is possible that some items were not relevant or that parents or AYAs did not feel connected with the clinicians conducting ACP conversations.

## Conclusions

The findings of this cohort study suggest that the PediSICP represents a significant step toward addressing the unmet need for pediatric ACP by providing a framework for clinicians caring for pediatric patients who are seriously ill and their families. The number of children living with serious illness is growing, which requires pediatric clinicians to become proficient in ACP to ensure the care they provide is concordant with patient and family goals and values.^43,66,67^ By equipping clinicians with the necessary training and tools, the PediSICP promotes meaningful ACP conversations that enhance clinician and family experiences and may improve therapeutic alliance and reduce anxiety. While findings of this pilot study are promising, future studies will evaluate the efficacy of the PediSICP on family-centered outcomes in a randomized controlled trial. Additionally, assessing the scalability and cost-effectiveness of the program will be essential for its widespread adoption.

## Notes

Supplement 1. **eTable.** Assessment of Parent Exploratory Outcome Measures Before and After the PediSICP Intervention

Supplement 2.
Data Sharing Statement
