# Supplementary material for: Feasibility of a Serious Illness Communication Program for Pediatric Advance Care Planning
Source: JAMA Netw Open. 2024 Jul 26;7(7):e2424626. doi: 10.1001/jamanetworkopen.2024.24626 (PMC11282445; doi:10.1001/jamanetworkopen.2024.24626)
Supplement: Supplement 1. — eTable. Assessment of Parent Exploratory Outcome Measures Before and After the PediSICP Intervention [file jamanetwopen-e2424626-s001.pdf]

Supplemental Online Content

DeCourcey DD, Bernacki RE, Nava-Coulter B, Lach S, Xiong N, Wolfe J. Feasibility of a serious illness communication program for pediatric advance care planning. *JAMA Netw Open*. 2024;7(7):e2424626. doi:10.1001/jamanetworkopen.2024.24626

eTable. Assessment of Parent Exploratory Outcome Measures Before and After the PediSICP Intervention

This supplemental material has been provided by the authors to give readers additional information about their work.

© 2024 DeCourcey DD et al. *JAMA Network Open*.

eTable. Assessment of Parent Exploratory Outcome Measures Before and After the *PediSICP* Intervention

Parent Exploratory Outcome Measures	Score Mean (SD)			P-Values[a]		
	Pre-Intervention	Post-Intervention	1mo Follow up	pre vs post	pre vs f/u	post vs f/u
Human Connection Scale	55.3 (7.8)	57.6 (6.4)	54.8 (8.7)	0.03	0.97	0.16
Collaborate-5	4.2 (0.6)	4.3 (0.6)	4.2 (0.7)	0.73	0.36	0.99
Quality of Communication Questionnaire	8.1 (1.6)	8.5 (1.6)	8.1 (1.7)	0.25	0.99	0.19
Generalized Anxiety Disorder-7 item	10.1 (7.3)	8.4 (6.9)	7.7 (6.8)	<0.01	0.03	0.48
Patient Health Questionnaire 9-item	7.0 (6.6)	7.4 (6.5)	7.9 (6.7)	0.76	0.62	0.98

[a]*Comparisons for continuous variables were performed using the Wilcoxon signed-rank test.*

© 2024 DeCourcey DD et al. *JAMA Network Open*.
