# Supplementary material for: Feasibility of a Serious Illness Communication Program for Pediatric Advance Care Planning
Source: JAMA Netw Open. 2024 Jul 26;7(7):e2424626. doi: 10.1001/jamanetworkopen.2024.24626 (PMC11282445; doi:10.1001/jamanetworkopen.2024.24626)
Supplement: Supplement 2. — Data Sharing Statement [file jamanetwopen-e2424626-s002.pdf]

Data Sharing Statement

DeCourcey. Feasibility of a Serious Illness Communication Program for Pediatric Advance Care Planning. *JAMA Netw Open*. Published July 26, 2024. doi:10.1001/jamanetworkopen.2024.24626

Data
Data available: No

Additional Information
Explanation for why data not available: This study includes protected information about minor-aged children and their parents who did not provide consent for all details/data to be shared and thus will be kept confidential.
